# Eating Spicy Food, Dietary Approaches to Stop Hypertension (DASH) Score, and Their Interaction on Incident Stroke in Southwestern Chinese Aged 30–79: A Prospective Cohort Study

**DOI:** 10.3390/nu15051222

**Published:** 2023-02-28

**Authors:** Liling Chen, Wenge Tang, Xiaomin Wu, Rui Zhang, Rui Ding, Xin Liu, Xiaojun Tang, Jing Wu, Xianbin Ding

**Affiliations:** 1 National Center for Chronic and Non-Communicable Disease Control and Prevention, Chinese Center for Disease Control and Prevention, Beijing 100050, China; 2 Institute of Chronic Non-Communicable Disease Control and Prevention, Chongqing Center for Disease Control and Prevention, Chongqing 400042, China; 3 Department of Epidemiology and Biostatistics, School of Public Health, Tianjin Medical University, Tianjin 300070, China; 4 First Medical College, Chongqing Medical University, Chongqing 400016, China; 5 School of Public Health and Management, Research Center for Medical and Social Development, Chongqing Medical University, Chongqing 400016, China

**Keywords:** spicy food, dietary approaches to stop hypertension, stroke incidence, interaction, prospective cohort study

## Abstract

Little is known about the association between spicy food intake, dietary approaches to stop hypertension (DASH) score, and incident stroke. This study aimed to explore the association of eating spicy food, DASH score, and their interaction with stroke incidence. We included 22,160 Han residents aged 30–79 in southwest China from the China Multi-Ethnic Cohort. Three hundred and twelve cases were newly diagnosed with stroke by October 8, 2022, during a mean of 45.5 months of follow-up. Cox regression analyses showed that eating spicy food reduced stroke risk by 34% among people with low DASH scores (HR 0.66, 95%CI 0.45–0.97), while individuals with high DASH scores versus low DASH scores had a 46% lower stroke incidence among spicy food nonconsumers (HR 0.54, 95%CI 0.36–0.82). The HR of the multiplicative interactive term was 2.02 (95%CI 1.24–3.30) and the overall estimates of relative excess risk due to interaction (RERI), attributable proportion due to interaction (AP), and the synergy index (S) were 0.54 (95%CI 0.24–0.83), 0.68 (95%CI 0.23–1.14), and 0.29 (95%CI 0.12–0.70), respectively. Consuming spicy food seems to be associated with lower stroke risk only in people who have a lower DASH score, while the beneficial effect of higher DASH scores seems to be found only among nonconsumers of spicy food, and a negative interaction may exist between them in southwestern Chinese aged 30–79. This study could provide scientific evidence for dietary guidance to reduce stroke risk.

## 1. Introduction

Stroke is the leading cause of death and disability-adjusted life-years (DALYs) in the world [1,2]. From 1990 to 2019, the absolute number of cases significantly increased with a 70.0% increase in incident strokes, an 85.0% increase in prevalent strokes, a 43.0% increase in deaths from stroke, and a 32.0% increase in DALYs due to stroke [2]. In China, the stroke incidence rate reached 276.7 per 100,000, and the mortality rate reached 153.9 per 100,000 in 2019 [3]. Therefore, the disease burden of stroke remains severe around the world and in China. Implementing effective prevention strategies is essential to mitigate the stroke burden.

A balanced diet, as one of the four cornerstones of health, has been widely studied for disease prevention and health improvement. Current internationally recommended dietary patterns tend to be based on a variety of healthy foods, and the benefits for cardiovascular disease are generally better than when controlling single isolated nutrients [4]. The Dietary Approaches to Stop Hypertension (DASH) diet is a well-accepted dietary pattern recommendation targeting blood pressure [5] and offers superior dietary guidance compared to an alternative Mediterranean diet for reducing cardiometabolic risks in less developed ethnic minority regions (LEMRs) [4]. In addition to lowering blood pressure, following the DASH diet has been found to improve cardiovascular disease (CVD) risk factors such as body weight, blood lipids, lipoproteins, inflammatory markers, and glucose–insulin homeostasis [6]. Previous research indicated that higher adherence to the DASH diet (i.e., a higher DASH score) is associated with lower stroke mortality (hazard ratios: 0.62, 0.97, respectively) [7,8] and incidence (hazard ratios: 0.61–0.88) [6,9,10].

Spicy food, defined mainly by chili pepper content, is widely consumed in many parts of the world [11,12,13]. The bioactive ingredients of spicy food, such as capsaicin, have been found to have antithrombotic and vasodilatory properties, anti-inflammatory properties, antioxidant properties, etc. [14,15]. A meta-analysis of four prospective cohort studies (from the United States, China, Italy, and Iran) showed that regularly consuming (≥ 1 day/week) spicy food was associated with a 12% lower risk of all-cause mortality [12]. Meta-analyses and prospective studies suggested that spicy food could reduce diastolic blood pressure (weighted mean differences of −1.90 mmHg) [16], reduce total cholesterol level (overall standardized mean difference: −0.52) [17], and reduce the risk of overweight/obesity (hazard ratio: 0.73–0.81) [18]. According to previous studies, spicy food has the ability to control metabolic syndrome and related disorders such as high blood pressure, obesity, and disordered lipid and glucose profiles [19,20], which are risk factors for stroke [2]. Therefore, we hypothesize that spicy food may have a protective effect on stroke onset.

Chongqing is located in southwest China, where the climate is humid and almost 50% of the residents consume spicy food daily [21,22]. The incidence of stroke is lower in Chongqing than the average level in China [23]. Is this lower rate related to consuming spicy food? Although the benefits of a higher DASH score for stroke are well established, the effects may vary across regions with different economic development levels, dietary habits, and lifestyles. How does it work in a region where spicy food is preferred? Reliable evidence from large-scale epidemiological studies on this topic is scarce. As spicy food and DASH score are both closely related to the daily diet and have cardiovascular-protective effects, we are interested in whether there is an interaction between them in terms of incident stroke. This study aimed to explore the association between spicy food intake, DASH score, and stroke incidence using the Han cohort aged 30–79 established by the China Multi-Ethnic Cohort (CMEC) study in Chongqing.

## 2. Methods

### 2.1. Study Population

**Figure 1:**
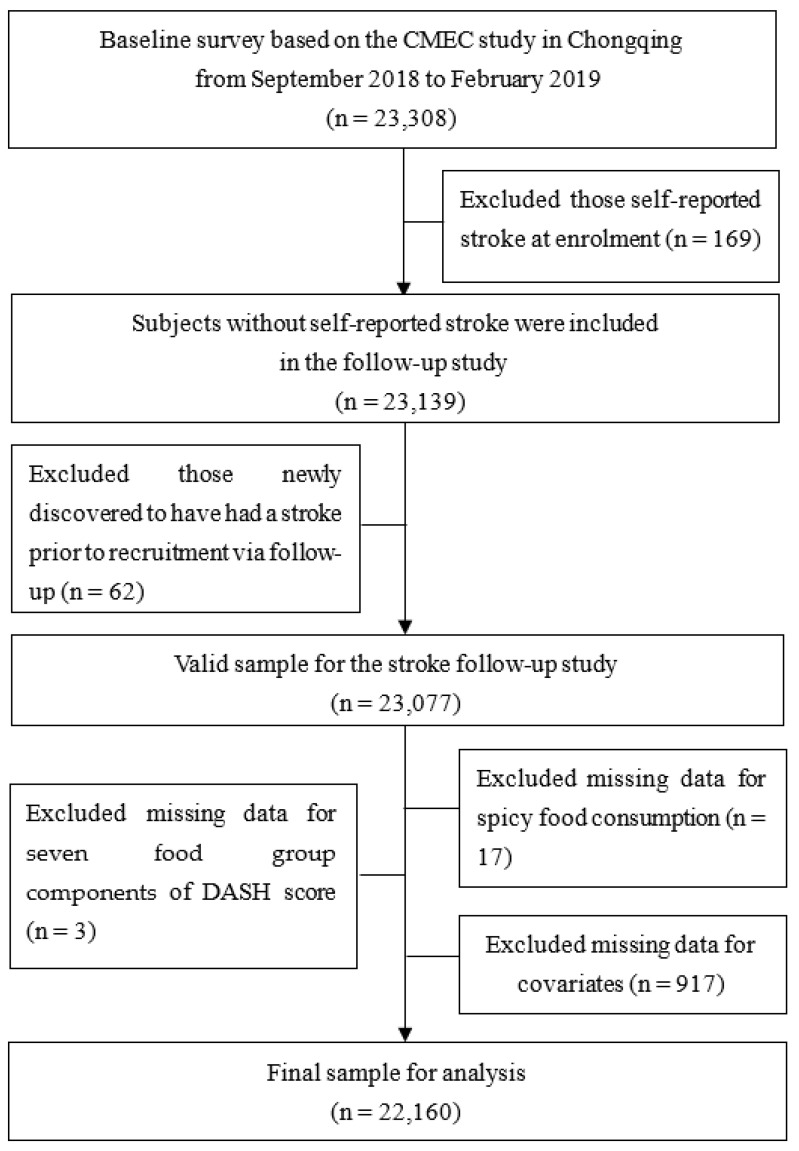
Data cleaning flowchart.

This is an ongoing community-based prospective study from Chongqing Municipality in southwest China, based on the China Multi-Ethnic Cohort (CMEC) study. Details of the CMEC study design have been described elsewhere [24]. In brief, a total of 23,308 Han Chinese participants, aged 30–79 years, who had lived in the local area for half a year or more, were recruited by multi-stage, stratified cluster sampling in consideration of both sex ratio and age ratio between September 2018 and February 2019. Baseline assessment consisted of an electronic questionnaire with face-to-face interviews (e.g., sociodemographics, diet and lifestyle, medical history), medical examinations (e.g., height, body weight, and blood pressure), and clinical laboratory tests (e.g., blood and urine specimens). Figure 1 shows the data cleaning flowchart. In this study, 22,160 participants were included in the final analysis. The inclusion criteria were: (1) completed the baseline (*n* = 23,308); (2) without self-reported stroke diagnosed by a physician at enrolment (*n* = 23,139). The exclusion criteria were: (1) newly discovered to have had a stroke prior to recruitment via follow-up (*n* = 62); (2) missing data for spicy food consumption (*n* = 17); (3) missing data for seven food group components of DASH score (*n* = 3); (4) missing data for covariates (*n* = 917). Ethic approvals from the Medical Ethics Committee of Chongqing Center for Disease Control and Prevention (2021(006),2017(001)) and the Sichuan University Medical Ethical Review Board (K2016038) were obtained, and informed consent was signed by all study participants.

### 2.2. Follow-Up and Outcome Assessment

Follow-up of the stroke was conducted in both passive and active modes. The passive follow-up was performed via linkage to the national cardiovascular event registration report system and the Chongqing death registry system. In active mode, a resurvey among 10% of surviving participants was conducted in late 2020 using the same measurements as the baseline survey. In addition, all survivors received telephone follow-ups every year since they entered the cohort. The main outcome examined in the present study was the incidence of stroke. According to the 10th version of the International Statistical Classification of Diseases (ICD-10), disease codes I60 to I64 were used to identify cases diagnosed with a stroke, including subarachnoid hemorrhage, intracerebral hemorrhage, ischemic stroke, and unclassified stroke, and excluding transient ischemic attack (TIA) and chronic cerebral arteriosclerosis [25]. Participants contributed person-months from their enrolment date until the onset of stroke event, death (from any cause), loss to follow-up, or the final follow-up assessment date (8 October 2022, for this current study), whichever came first.

### 2.3. Assessment of Spicy Food Consumption and DASH Score

Food intake was collected by a quantitative food frequency questionnaire (FFQ) of the CMEC study, and its validity and reproducibility were both assessed by conducting repeated FFQ and 24 h dietary recalls (24 HDRs) in a resurvey in 2020 [5].

As with the China Kadoorie Biobank study [26], spicy food intake refers to the consumption of any “hot” spices when cooking or eating, including fresh or dried chili pepper, chili sauce, chili oil, or other hot spices. Participants were asked about their consumption frequency (never or rarely, only occasionally, 1–2 days/week, 3–5 days/week, or 6–7 days/week) in the past month at baseline. Those who selected the last 3 categories (1–2 days/week, 3–5 days/week, or 6–7 days/week) were defined as regular spicy food consumers and nonconsumers (never or rarely, only occasionally) as the reference group in this study.

A modified DASH diet was verified to be more effective at reducing cardiovascular and cerebrovascular risks in a randomized controlled trial [27]. To assess adherence to the modified DASH diet, we calculated the DASH score following scoring methods developed by Chiu et al. [27] and Fung et al. [28] with slight adaption according to the CEMC data [4]. Each of the seven food groups, including whole grains, fresh fruits, vegetables, beans, red meat products, dairy, and sodium, was assigned a score of 1 to 5 based on the quintile of the average food intake. For whole grains, fresh fruits, vegetables, beans, and dairy, a score of 5 was given for the highest quintile, while a score of 1 was given for the lowest quintile. For red meat products and sodium, this pattern of scoring was inverted. The sum of the seven component scores resulted in an overall DASH score ranging from 7 (minimal adherence) to 35 (maximal adherence). Based on the lowest tertile of DASH score as the cutoff point, all participants were categorized into two groups (low DASH score: <19; high DASH score: ≥19), with the low DASH score group as the reference.

Spicy food consumption (no and yes) and DASH score (low DASH score and high DASH score) were both dichotomous factors, in which case we have four combinations by combining them, i.e., “Not spicy/Low DASH score”, “Not spicy/High DASH score”, “Spicy/Low DASH score”, and “Spicy/High DASH score”, using “Not spicy/Low DASH score” as reference.

### 2.4. Assessment of Covariates

Sociodemographic covariates included gender (male and female), age (<60 years and ≥60 years), and education level (primary school and below, middle school, high school, college or university and above). Smoking status and drinking status were both classified as no and yes. Physical activity was estimated by summing up the corresponding metabolic equivalent values (METs) of four domains including leisure, work, transportation, and housework [29]. According to the tertiles, physical activity was categorized into <21.94 METs h/day, 21.94–35.55 METs h/day, and >35.55 METs h/day. Body mass index (BMI) was calculated using weight in kilograms divided by the square of height in meters (kg/m^2^) and grouped into <18.5 kg/m^2^, 18.5–23.9 kg/m^2^, 24–27.9 kg/m^2^, and ≥28 kg/m^2^ according to the weight criteria for adults in China [30]. Hypertension was defined as an average of three measurements for systolic or diastolic blood pressure (SBP/DBP) ≥140/90 mmHg or a self-reported diagnosis of hypertension by a physician [31]. Participants having any one of the following conditions were regarded as having dyslipidemia: (1) triacylglycerol (TG) ≥2.26 mmol/l; (2) serum total cholesterol (TC) ≥6.22 mmol/L; (3) low-density lipoprotein cholesterol (LDLC) ≥4.14 mmol/L; (4) and high-density lipoprotein cholesterol (HDLC) <1.04 mmol/L; (5) a self-reported diagnosis of hyperlipemia by a physician [32]. Diabetes was diagnosed as fasting blood glucose (FBG) ≥7.0 mmol/L, glycosylated hemoglobin percentage of ≥6.5%, or self-reported diagnosis of diabetes by a physician [33]. A family history of stroke-related diseases was defined as a self-reported family history of hypertension, diabetes, stroke, or acute myocardial infarction (AMI). In addition to the 11 covariates listed above, spicy food and DASH scores were adjusted for each other.

### 2.5. Statistical Analysis

Continuous data were described as the mean ± SD, and statistical significance was assessed by the independent samples *t*-test. Categorical variables were described as numbers (percentages), and statistical significance was assessed by the chi-square test.

Cox proportion hazard regression models were applied to calculate the hazard ratios (HRs) and confidence intervals (CIs) of spicy food consumption, DASH score, and their interaction with stroke incidence. Subgroup analyses by spicy food consumption and DASH score were performed to examine the potential interaction. The relative excess risk due to interaction (RERI), the attributable proportion due to interaction (AP), and the synergy index (S) were used to assess the additive interaction between spicy food and DASH score [34]. These measures were output according to the following equations: (1) RERI = HR_11_-HR_10_-HR_01_ + 1; (2) AP = RERI/HR_11_; (3) S = (HR_11_-1)/[(HR_10_-1) + (HR_01_-1)]. In this study, with “Not spicy/Low DASH score” (HR_00_ = 1) as the reference category, HR_01_, HR_10_, and HR_11_ were the hazard ratios for groups of “Not spicy/High DASH score”, “Spicy/Low DASH score”, and “Spicy/High DASH score”, respectively. If there is no biological interaction, RERI and AP are equal to 0 and S is equal to 1.

All analyses were performed using SPSS (Version 25.0. IBM Corp., Armonk, NY, USA) and Excel 2010 (Microsoft Office 2010, Microsoft Corporation, USA). A two-sided *p*-value < 0.05 was considered statistically significant.

## 3. Results

### 3.1. General Characteristics

Among the 21,160 participants, the average age was 51.4 ± 11.7 years and 53.2% were females (Table 1). Seventy-six percent of them consumed spicy food in the past month and 69.5% had high DASH scores. Participants who ate spicy food were more likely to be males, younger, smokers, drinkers, and have a family history of stroke-related diseases; to have higher education levels, physical activity levels, BMI levels, DASH score, and dyslipidemia prevalence; and to have lower hypertension and diabetes prevalence than those who did not eat spicy food (all *p* < 0.05). Conversely, compared to those with low DASH scores, individuals with high DASH scores were more likely to be females, nonsmokers, and spicy food consumers; and have lower BMI levels and dyslipidemia prevalence (all *p* < 0.05).

### 3.2. Independent Effect of Spicy Food Consumption and DASH Score

The mean follow-up time was 45.5 (4.0) months, and 312 individuals (1.4%) were diagnosed with stroke during the follow-up period, equivalent to an incidence rate of 371.68 per 100,000 person-years (Table 2). In the crude Cox regression model, except for the elderly (all *p* > 0.05), both spicy food consumption and DASH score were inversely associated with stroke risk in the overall population and among both males and females, and those <60 years of age (all *p* < 0.05). However, after adjusting for covariates in model 1, neither spicy food consumption nor DASH score was significantly correlated with stroke incidence (all *p* > 0.05).

**Figure 2:**
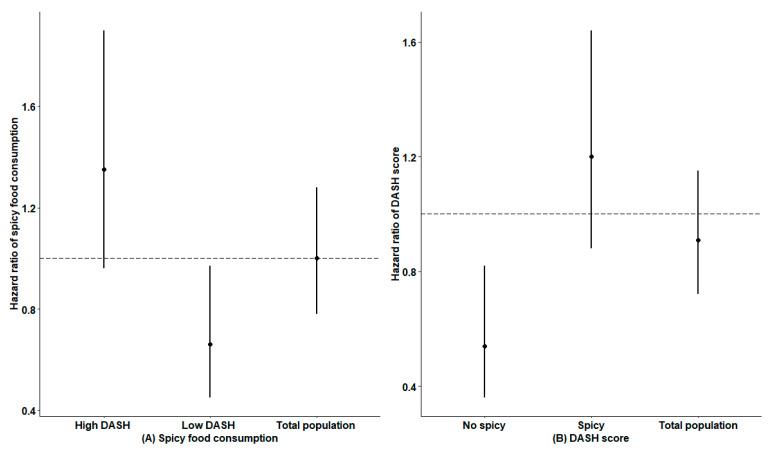
Subgroup analyses by DASH score or spicy food consumption. (**A**) The association between spicy food consumption and incident stroke, stratified by DASH score. (**B**) The association between DASH score and incident stroke, stratified by spicy food consumption.

Separate subgroup analyses by spicy food consumption and DASH scores implied that there may be an interaction between them (Figure 2). Figure 2A shows that eating spicy food reduced the risk of developing stroke by 34% among people with low DASH scores (HR 0.66, 95%CI 0.45–0.97, *p* = 0.03), whereas the protective effect was not found among people with high DASH scores (*p* > 0.05). After stratifying by spicy food consumption (Figure 2B), individuals with high DASH scores versus low DASH scores had a 46% decrease in stroke incidence among spicy food nonconsumers (HR 0.54, 95%CI 0.36–0.82, *p* < 0.01). However, similarly, this inverse association was not discovered among those who consumed spicy food (*p* > 0.05).

### 3.3. Interaction Effect of Spicy Food Consumption and DASH Score on Stroke Incidence

In model 2 (Table 3), the interaction term was created by combining spicy food consumption and DASH score. Compared to the group of ”Not spicy/Low DASH score”, participants in the group of “Not spicy/High DASH score” had the lowest risk of stroke (HR_01_ 0.58, 95%CI 0.39–0.86, *p* < 0.01), followed by the group of “Spicy/Low DASH score” (HR_10_ 0.67, 95%CI 0.46–0.97, *p* = 0.03), but little difference was found in the group of “Spicy/High DASH score” (HR_11_ 0.78, 95%CI 0.57–1.08, *p* = 0.14). This interaction term was also found to be significantly associated with incident stroke among males (HR_01_ = 0.57, 95%CI 0.39–0.86, *p* < 0.01; HR_10_ = 0.62, 95%CI 0.39–1.01, *p* = 0.05) and those < 60 years of age (HR_01_ 0.43, 95%CI 0.19–1.00, *p* = 0.05; HR_10_ 0.43, 95%CI 0.22–0.86, *p* = 0.02; HR_11_ 0.44, 95%CI 0.237–0.831, *p* = 0.01). Similar trends were also observed among females and those ≥ 60 years of age (*p* > 0.05).

In model 3 (Table 3), a multiplicative interaction effect between spicy food consumption and DASH score was revealed. The corresponding HRs of the multiplicative interaction term for the overall population and males as well as the elderly were 2.02 (95%CI 1.24–3.30, *p* = 0.01), 1.99 (95%CI 1.03–3.85, *p* = 0.04), and 1.89 (95%CI 1.07–3.34, *p* = 0.03), respectively (Table 3). HRs of the multiplicative interaction term among females and those < 60 years of age were both marginally significant (*p* > 0.05).

Based on adjusted HR_10_, HR_01_, and HR_11_, additive interaction indicators were calculated and showed a negative additive interaction between spicy food consumption and DASH score (Table 4). RERIs of the overall population, males, females, those <60 years of age, and the elderly were 0.54 (95%CI 0.24–0.83), 0.52 (95%CI 0.13–0.90), 0.52 (95%CI 0.02–1.02), 0.58 (95%CI 0.15–1.00), and 0.51 (95%CI 0.11–0.90), respectively. APs were observed to be statistically significant in the overall population, and among males and the elderly, with corresponding measures of 0.68 (95%CI 0.23–1.14), 0.73 (95%CI 0.08–1.38), 0.54 (95%CI 0.06–1.02), respectively. S estimates were 0.29 (95%CI 0.12–0.70) in the overall population, 0.36 (0.17–0.78) for males, and 0.50 (0.34–0.74) for those <60 years of age.

### 3.4. Summary of Main Results

**Figure 3:**
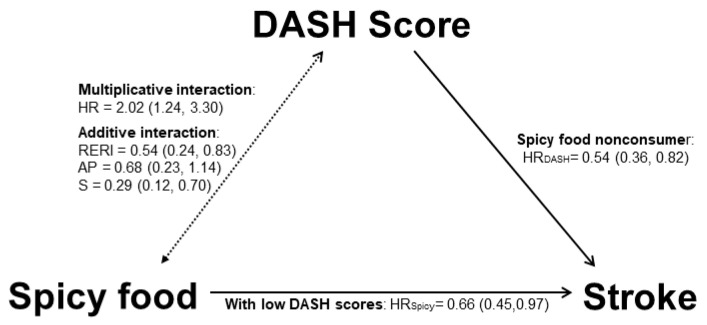
The association between spicy food consumption, a higher DASH score, and incident stroke among southwestern Chinese aged 30–79 years. HR_DASH_: the HR of a higher DASH score on incident stroke among nonconsumers of spicy food; HR_Spicy_: the HR of eating spicy food on incident stroke among people with low DASH scores.

Figure 3 summarizes the association between spicy food consumption, a higher DASH score, and incident stroke in the current study. A negative interaction between spicy food consumption and DASH score was suggested by both multiplicative interaction (HR 2.02, 95%CI 1.24–3.30) and additive interaction (RERI 0.54, 95%CI 0.24–0.83; AP 0.68, 95%CI 0.23–1.14; S 0.29, 95%CI 0.12–0.70). It was found that consuming spicy food reduced the risk of developing stroke by 34% among people with low DASH scores but not among those with high DASH scores. A higher DASH score was linked to a 46% lower stroke incidence for spicy food nonconsumers but not for spicy food consumers.

## 4. Discussion

In this study, we found that consuming spicy food reduced the risk of developing stroke by 34% among people with low DASH scores, whereas the protective effect was not found among those with high DASH scores. A higher DASH score was linked to a 46% lower stroke incidence among those who did not regularly consume spicy food, whereas the protective effect was not found among spicy food consumers. The beneficial effect of a higher DASH score appears to be greater than that of spicy food intake for incident stroke and a negative interaction was discovered between them in terms of multiplicative interaction as well as additive interaction.

### 4.1. Spicy Food May Be an Effective Nutritional Strategy for Preventing Stroke

To our knowledge, this is the first prospective study assessing the association between spicy food and stroke incidence. Spicy food consumption was found to be linked with lower stroke risk in people with low DASH scores, whereas the protective effect was not found among those with high DASH scores. In line with our results, Bonaccio et al. [35] demonstrated that using chili pepper was associated with a lower risk of cerebrovascular mortality in an Italian cohort comprising 23,811 participants aged more than 35 years old. In 2017, the U.S. National Health and Nutrition Examination Survey (NHANES) trial [36] reported a lower risk of total mortality associated with the consumption of hot chili peppers, and similar trends of reduction (statistically nonsignificant) were seen for death from stroke. However, a Chinese population-based study (*n* = 487,375, aged 30–79 years) [37] found no statistically significant link between eating spicy food daily and lower stroke mortality. It may be attributed to some risk factors that were not adjusted in the final analysis, such as salt intake, which is high in China and could increase stroke risk [38].

In this study, among residents consuming spicy food in Chongqing, about 98.8% of them consumed chili peppers. Capsaicin, one of the main components of chili peppers, was confirmed to have cardiovascular-protecting effects by activating transient potential vanilloid 1 (TRPV 1) and substance *p* and the release of calcitonin gene-related peptide (CGRP) [11,14]. Moreover, capsaicin can prevent platelet aggregation and the activity of clotting factors VIII and IX, resulting in decreasing stroke events [14,39]. Additionally, spicy food may reduce stroke risk by decreasing metabolic risk factors such as hypertension, obesity, hyperglycemia, and hyperlipidemia [14,40]. Thus, it is also biologically plausible that spicy food may be an effective nutritional strategy for preventing stroke, especially in people who have a lower DASH score.

### 4.2. A Higher DASH Score Plays a Beneficial Role in Incident Stroke

In this study, it was found that a higher DASH score, indicating greater adherence to the DASH diet, was associated with a lower stroke incidence among spicy food nonconsumers, whereas the protective effect was not found among spicy food consumers. As far as we know, few studies have considered spicy food consumption when analyzing the association between DASH score and stroke onset. Previous studies still found a protective effect of adherence to the DASH diet for incident stroke, although they did not adjust for spicy food consumption [8,9,10]. It may be because the proportion of respondents eating spicy food in these studies was not as high as ours in Chongqing which is famous for its spicy food preference [41,42].

The DASH diet is rich in fruits, vegetables, whole grains, legumes, and dairy products, and high consumption of them either together or alone was confirmed to be inversely associated with stroke and CVD incidence by systematic reviews and meta-analyses of prospective cohort studies, while the restricted red meat and dietary sodium in DASH diets were positively correlated with the increased incidence of stroke and CVD [8,38]. Further, dietary fiber and other essential nutrients richly found in the DASH diet, such as magnesium, potassium, and phytochemicals, have anti-inflammatory and antioxidant activity and reduce angiogenesis, which has stroke benefits [43,44,45]. In addition, adherence to the DASH diet was identified to improve blood pressure, lipids, body weight, and blood glucose, which is also beneficial for the prevention and control of stroke [8,46]. Therefore, it is biologically tenable that a higher DASH score reduces stroke risk in people who do not regularly eat spicy food. However, the reason for the lack of protective effect among consumers of spicy food needs to be studied further.

### 4.3. A Negative Interaction May Exist between Spicy Food Consumption and DASH Score

As far as we know, the interaction between spicy food consumption and DASH score on stroke incidence was found for the first time. Subgroup analyses showed that consuming spicy food is associated with a lower stroke incidence only in people who have a lower DASH score, while the beneficial effect of a higher DASH score is found only among nonconsumers of spicy food. Compared to the group of ”Not spicy/Low DASH score”, people in the group of “Not spicy/High DASH score” had the lowest risk of stroke, followed by the group of “Spicy/Low DASH score”, and then the group of “Spicy/High DASH score”. Moreover, the HR of the multiplicative interaction term was significantly greater than 1, whereas those of spicy food and DASH score were both less than 1. Regarding additive indicators, both RERIs and APs were more than 0, and S estimates were less than 1. All these results demonstrated that a negative interaction may exist between spicy food consumption and DASH score.

Why is the interaction negative? Little is known about this. Perhaps spicy food and the DASH diet both contain certain nutrients, such as magnesium or potassium. The beneficial effects may diminish once the shared nutrients reach a specific amount when people consume spicy food and follow a DASH diet. In addition, some of the active ingredients in spicy food and the DASH diet may be antagonistic to each other. These speculations need to be explored in further studies. In this study, the fact that the distribution of some risk factors differed between spicy food intake and DASH score may also provide some clues: people with high DASH scores were more likely to be females, nonsmokers, and to have lower BMI levels and dyslipidemia prevalence, whereas those characteristics were distributed conversely for those who consumed spicy food. The incidence of stroke is always found to be higher for males and smokers, and higher BMI and dyslipidemia are the common risk factors for stroke [2]. Additionally, some overlaps may exist between spicy food and DASH score. The main source of spicy food in this population is chili pepper (about 98.8%) which would also be captured in the vegetable section of the DASH score. However, this overlap would be limited because vegetables refer to many categories and the intake of other vegetables is usually much greater than that of chili peppers. The correlation coefficient (r = 0.10) between spicy food intake and DASH score in this study is small, also suggesting that the overlap is limited. In brief, a negative interaction may exist between spicy food consumption and DASH score. Further research is needed on the mechanisms of this interaction.

### 4.4. Strengths and Limitations

To our knowledge, this is the first prospective cohort study to comprehensively examine the interaction between spicy food consumption and DASH score on stroke incidence, including both the multiplicative and additive interactions. Furthermore, our study explored the association of spicy food consumption with the risk of developing stroke, which has been rarely performed. Nonetheless, limitations are worth noting. First, due to the preponderance of ischemic stroke versus hemorrhagic stroke and short follow-up time, the number of participants with specific types of stroke was insufficient to conduct separate analyses, but preliminary analyses showed similar results between the two types of stroke (data not shown). Second, the dietary data were self-reported at baseline, likely leading both to measurement error and the possibility of unaccounted changes in dietary behavior before the incident stroke. Furthermore, the FFQ used in this study did not provide information in sufficient detail to allow for computing other indices, such as the dietary inflammatory index [47], which would help to pinpoint the mechanisms of action. Third, exposure factors were only roughly grouped according to whether participants ate spicy food and whether their DASH score was ≥19, thus the dose-response relationship between exposure and outcome needs further research. Fourth, these results were obtained among Han ethnicity residents aged 30–79 years, from areas eating spicy foods relatively often, so extrapolating the findings to other areas or populations should be noted.

## 5. Conclusions

In conclusion, consuming spicy food seems to be associated with a lower stroke incidence only in people who have a lower DASH score, while the beneficial effect of a higher DASH score seems to be found only among nonconsumers of spicy food. The beneficial effect of a higher DASH score appears to be greater than that of spicy food consumption for incident stroke, and a negative interaction may exist between them in southwestern Chinese aged 30–79. This study could provide scientific evidence for dietary guidance to reduce stroke risk. Future studies, such as using enhanced dietary assessment methods to compute the dietary inflammatory index that helps determine whether spicy food or the DASH score works through inflammation-related pathways, are required.

## Figures and Tables

**Table 1:** Baseline characteristics of study participants by spicy food consumption and DASH score.

Variables	Total (*n* = 22,160)	Spicy Food Consumption			DASH Score		
		No (*n* = 5322)	Yes (*n* = 16,838)	*p*	Low (*n* = 6755)	High (*n* = 15,405)	*p*
Age (years)	51.4 ± 11.7	54.7 ± 12.2	50.4 ± 11.4	<0.01	52.6 ± 11.5	50.9 ± 11.8	0.03
Age group (years)				<0.01			<0.01
<60	16,198 (73.1%)	3299 (62.0%)	12,899 (76.6%)		4741 (70.2%)	11,457 (74.4%)	
≥60	5962 (26.9%)	2023 (38.0%)	3939 (23.4%)		2014 (29.8%)	3948 (25.6%)	
Gender				<0.01			<0.01
Male	10,371 (46.8%)	2354 (44.2%)	8017 (47.6%)		3811 (56.4%)	6560 (42.6%)	
Female	11,789 (53.2%)	2968 (55.8%)	8821 (52.4%)		2944 (43.6%)	8845 (57.4%)	
Education level				<0.01			<0.01
Primary school and below	7197 (32.5%)	2303 (43.3%)	4894 (29.1%)		3194 (47.3%)	4003 (26.0%)	
Middle school	7154 (32.3%)	1609 (30.2%)	5545 (32.9%)		2062 (30.5%)	5092 (33.1%)	
High school	4108 (18.5%)	735 (13.8%)	3373 (20.0%)		818 (12.1%)	3290 (21.4%)	
College or university and above	3701 (16.7%)	675 (12.7%)	3026 (18.0%)		681 (10.1%)	3020 (19.6%)	
Smoking				<0.01			<0.01
No	16,220 (73.2%)	4325 (81.3%)	11,895 (70.6%)		4513 (66.8%)	11,707 (76.0%)	
Yes	5940 (26.8%)	997 (18.7%)	4943 (29.4%)		2242 (33.2%)	3698 (24.0%)	
Drinking				<0.01			<0.01
No	10,115 (45.6%)	3360 (63.1%)	6755 (40.1%)		3320 (49.1%)	6795 (44.1%)	
Yes	12,045 (54.4%)	1962 (36.9%)	10,083 (59.9%)		3435 (50.9%)	8610 (55.9%)	
Physical activity (METs h/day)				<0.01			<0.01
<21.94	7260 (32.8%)	2166 (40.7%)	5094 (30.3%)		2170 (32.1%)	5090 (33.0%)	
21.94–35.55	7459 (33.7%)	1544 (29.0%)	5915 (35.1%)		2014 (29.8%)	5445 (35.3%)	
>35.55	7441 (33.6%)	1612 (30.3%)	5829 (34.6%)		2571 (38.1%)	4870 (31.6%)	
BMI (Body mass index, kg/m^2^)				<0.01			<0.01
18.5 ≤ BMI< 24.0	9476 (42.8%)	2371 (44.6%)	7105 (42.2%)		2649 (39.2%)	6827 (44.3%)	
BMI < 18.5	338 (1.53%)	87 (1.63%)	251 (1.49%)		93 (1.38%)	245 (1.59%)	
24.0 ≤ BMI < 28	9107 (41.1%)	2164 (40.7%)	6943 (41.2%)		2854 (42.3%)	6253 (40.6%)	
BMI ≥ 28	3239 (14.6%)	700 (13.2%)	2539 (15.1%)		1159 (17.2%)	2080 (13.5%)	
Hypertension				<0.01			<0.01
No	14,422 (65.1%)	3226 (60.6%)	11,196 (66.5%)		3979 (58.9%)	10,443 (67.8%)	
Yes	7738 (34.9%)	2096 (39.4%)	5642 (33.5%)		2776 (41.1%)	4962 (32.2%)	
Dyslipidemia				0.02			<0.01
No	15,111 (68.2%)	3697 (69.5%)	11,414 (67.8%)		4470 (66.2%)	10,641 (69.1%)	
Yes	7049 (31.8%)	1625 (30.5%)	5424 (32.2%)		2285 (33.8%)	4764 (30.9%)	
Diabetes				<0.01			<0.01
No	19,577 (88.3%)	4613 (86.7%)	14,964 (88.9%)		5900 (87.3%)	13,677 (88.8%)	
Yes	2583 (11.7%)	709 (13.3%)	1874 (11.1%)		855 (12.7%)	1728 (11.2%)	
DASH score				<0.01			-
Low	6755 (30.5%)	2065 (38.8%)	4690 (27.9%)		-	-	
High	15,405 (69.5%)	3257 (61.2%)	12,148 (72.1%)		-	-	
Spicy food consumption				-			<0.01
No	5322 (24.0%)	-	-		2065 (30.6%)	3257 (21.1%)	
Yes	16,838 (76.0%)	-	-		4690 (69.4%)	12,148 (78.9%)	
Family history of stroke-related diseases				<0.01			<0.01
No	11,834 (53.4%)	3169 (59.5%)	8665 (51.5%)		3898 (57.7%)	7936 (51.5%)	
Yes	10,326 (46.6%)	2153 (40.5%)	8173 (48.5%)		2857 (42.3%)	7469 (48.5%)	

Note: Continuous data were described as the mean ± SD, and statistical significance was assessed by the independent samples *t*-test. Categorical data were summarized as percentages (%), and statistical significance was assessed by a chi-square test. DASH: dietary approaches to stop hypertension, METs: metabolic equivalent values, BMI: body mass index.

**Table 2:** Association of spicy food consumption and DASH score with stroke risk.

Variables	Number of Events/*n*	Incidence Density (1/100,000 Person-Years)	Crude Model		Model 1	
			HR (95%CI)	*p*	HR (95%CI)	*p*
**Total population**	312 */22,160	**371.68**				
Spicy food consumption						
No	103/5322	515.68	1.00		1.00	
Yes	209/16,838	326.72	0.64 (0.50, 0.80)	< 0.01	1.00 (0.78, 1.28)	1.00
DASH score						
Low	117/6755	459.40	1.00		1.00	
High	195/15,405	333.48	0.73 (0.58, 0.92)	< 0.01	0.91 (0.72, 1.15)	0.43
**Males ^a^**	**172/10,371**	**440.64**				
Spicy food consumption						
No	58/2354	664.64	1.00		1.00	
Yes	114/8017	376.14	0.57 (0.41, 0.78)	< 0.01	0.90 (0.64, 1.25)	0.52
DASH score						
Low	72/3811	503.49	1.00		1.00	
High	100/6560	404.30	0.81 (0.60, 1.09)	0.16	0.89 (0.65, 1.23)	0.48
**Females ^a^**	**140/11,789**	**311.75**				
Spicy food consumption						
No	45/2968	400.11	1.00		1.00	
Yes	95/8821	282.22	0.71 (0.50, 1.01)	0.05	1.13 (0.78, 1.63)	0.52
DASH score						
Low	45/2944	402.94	1.00		1.00	
High	95/8845	281.56	0.70 (0.49, 1.00)	0.05	0.95 (0.65, 1.37)	0.78
**Age < 60 years ^b^**	**82/16,198**	**132.77**				
Spicy food consumption						
No	25/3299	199.84	1.00		1.00	
Yes	57/12,899	115.73	0.58 (0.36, 0.93)	0.02	0.67 (0.41, 1.09)	0.11
DASH score						
Low	34/4741	188.71	1.00		1.00	
High	48/11,457	109.73	0.58 (0.38, 0.90)	0.02	0.78 (0.49, 1.25)	0.31
**Age ≥ 60 years ^b^**	**230/5962**	**1036.95**				
Spicy food consumption						
No	78/2023	1045.10	1.00		1.00	
Yes	152/3939	1032.82	0.99 (0.76, 1.30)	0.95	1.127 (0.849, 1.496)	0.41
DASH score						
Low	83/2014	1113.89	1.00		1.00	
High	147/3948	998.03	0.90 (0.69, 1.18)	0.44	0.96 (0.73, 1.27)	0.79

Crude model: without adjustment; Model 1: adjusted for gender (male, female), age (<60 years and ≥60 years), education level (primary school and below, middle school, high school, college or university and above), smoking (no and yes), drinking (no and yes), physical activity (<21.94 METs h/day, 21.94–35.55 METs h/day, and >35.55 METs h/day), BMI (18.5–24.0 kg/m^2^, <18.5 kg/m^2^, 24.0–28 kg/m^2^, and ≥28 kg/m^2^), hypertension (no and yes), dyslipidemia (no and yes), diabetes (no and yes), family history of stroke-related diseases (no and yes), spicy food consumption (only with DASH score as the exposure variable), DASH score (only with spicy food consumption as the exposure variable); * subarachnoid hemorrhage (*n* = 14); intracerebral hemorrhage (*n* = 46); ischemic stroke (*n* = 246); unclassified stroke (*n* = 6); ^a^ without adjustment for gender; ^b^ without adjustment for age.

**Table 3:** Association of the interaction between spicy food consumption and DASH score with stroke risk.

Variables	Number of Events/*n*	Incidence Density (1/100,000 Person-Years)	Crude Model		Model 2		Model 3	
			HR (95%CI)	*p*	HR (95%CI)	*p*	HR (95%CI)	*p*
**Total population**	**312/22,160**	**371.68**						
Interaction *							2.02 (1.24, 3.30)	<0.01
Not spicy/Low DASH score	57/2065	742.86	1.00		1.00		-	-
Not spicy/High DASH score	46/3257	373.97	0.51 (0.34, 0.75)	<0.01	0.58 (0.39, 0.86)	0.01	-	-
Spicy/Low DASH score	60/4690	337.17	0.46 (0.32, 0.65)	<0.01	0.67 (0.46, 0.97)	0.03	-	-
Spicy/High DASH score	149/12,148	322.69	0.44 (0.32, 0.59)	<0.01	0.78 (0.57, 1.08)	0.14	-	-
**Males ^a^**	**172/10,371**	**440.64**						
Interaction *							1.99 (1.03, 3.85)	0.04
Not spicy/Low DASH score	35/1088	874.71	1.00		1.00		-	-
Not spicy/High DASH score	23/1266	486.75	0.56 (0.33, 0.95)	0.03	0.57 (0.33, 0.98)	0.04	-	-
Spicy/Low DASH score	37/2723	359.26	0.41 (0.26, 0.66)	<0.01	0.62 (0.39, 1.01)	0.05	-	-
Spicy/High DASH score	77/5294	384.83	0.44 (0.30, 0.66)	<0.01	0.71 (0.46, 1.09)	0.11	-	-
**Females ^a^**	**140/11,789**	**311.75**						
Interaction *							1.90 (0.89, 4.02)	0.10
Not spicy/Low DASH score	22/977	599.17	1.00		1.00		-	-
Not spicy/High DASH score	23/1991	303.62	0.51 (0.28, 0.91)	0.02	0.64 (0.35, 1.15)	0.14	-	-
Spicy/Low DASH score	23/1967	306.83	0.51 (0.29, 0.92)	0.03	0.76 (0.42, 1.37)	0.35	-	-
Spicy/High DASH score	72/6854	275.17	0.46 (0.29, 0.74)	<0.01	0.91 (0.55, 1.51)	0.72	-	-
**Age < 60 years ^b^**	**82/16,198**	**132.77**						
Interaction *							2.38 (0.88, 6.41)	0.09
Not spicy/Low DASH score	16/1222	347.72					-	-
Not spicy/High DASH score	9/2077	113.80	0.33 (0.15, 0.74)	0.01	0.43 (0.19, 1.00)	0.05	-	-
Spicy/Low DASH score	18/3519	134.18	0.39 (0.20, 0.76)	0.01	0.43 (0.22, 0.86)	0.02	-	-
Spicy/High DASH score	39/9380	108.83	0.32 (0.18, 0.56)	<0.01	0.44 (0.24, 0.83)	0.01	-	-
**Age ≥ 60 years ^b^**	**230/5962**	**1036.95**						
Interaction *							1.89 (1.07, 3.34)	0.03
Not spicy/Low DASH score	41/843	1334.78	1.00		1.00		-	-
Not spicy/High DASH score	37/1180	842.49	0.63 (0.41, 0.99)	0.04	0.65 (0.41, 1.02)	0.06	-	-
Spicy/Low DASH score	42/1171	958.98	0.72 (0.47, 1.11)	0.14	0.77 (0.50, 1.20)	0.25	-	-
Spicy/High DASH score	110/2768	1064.10	0.80 (0.56, 1.15)	0.23	0.95 (0.65, 1.38)	0.78	-	-

* The interaction terms in model 2 (including four combinations by combining spicy food consumption and DASH score) and model 3 (a multiplicative term) were different; model 2: the interaction term was created by combining spicy food consumption and DASH score; adjusted for gender (male, female), age (<60 years and ≥60 years), education level (primary school and below, middle school, high school, college or university and above), smoking (no and yes), drinking (no and yes), physical activity (<21.94 METs h/day, 21.94–35.55 METs h/day, and >35.55 METs h/day), BMI (18.5–24.0 kg/m^2^, <18.5 kg/m^2^, 24.0–28 kg/m^2^, and ≥28 kg/m^2^), hypertension (no and yes), dyslipidemia (no and yes), diabetes (no and yes), family history of stroke-related diseases (no and yes); model 3: the interaction term was the multiplicative term (spicy food consumption × DASH score); adjusted for the same covariates as model 2; ^a^ without adjustment for gender; ^b^ without adjustment for age.

**Table 4:** Additive interaction measures between spicy food consumption and DASH score.

	RERI (95%CI)	AP (95%CI)	S (95%CI)
Total population	0.54 (0.24, 0.83)	0.68 (0.23, 1.14)	0.29 (0.12, 0.70)
Males	0.52 (0.13, 0.90)	0.73 (0.08, 1.38)	0.36 (0.17, 0.78)
Females	0.52 (0.02, 1.02)	0.57 (−0.08, 1.22)	0.14 (0.002, 12.65)
Age < 60 years	0.58 (0.15, 1.00)	1.33 (−0.11, 2.77)	0.50 (0.34, 0.74)
Age ≥ 60 years	0.51 (0.11, 0.90)	0.54 (0.06, 1.02)	0.10 (0.0003, 31.44)

RERI: the relative excess risk due to interaction, AP: the attributable proportion due to interaction, S: the synergy index.

## Data Availability

Our study relied on data from the China Multi-Ethnic Cohort study. The summary dataset used and analyzed during the current study is available from the corresponding author upon reasonable request.

## References

[1] Feigin V.L., Brainin M., Norrving B., Martins S., Sacco R.L., Hacke W., Fisher M., Pandian J., Lindsay P. (2022). World Stroke Organization (WSO): Global Stroke Fact Sheet 2022. Int. J. Stroke.

[2] GBD 2019 Stroke Collaborators (2021). Global, regional, and national burden of stroke and its risk factors, 1990-2019: A systematic analysis for the Global Burden of Disease Study 2019. Lancet Neurol..

[3] Ma Q., Li R., Wang L., Yin P., Wang Y., Yan C., Ren Y., Qian Z., Vaughn M.G., McMillin S.E. (2021). Temporal trend and attributable risk factors of stroke burden in China, 1990-2019: An analysis for the Global Burden of Disease Study 2019. Lancet Public Health.

[4] Xiao X., Qin Z., Lv X., Dai Y., Ciren Z., Yangla Y., Zeng P., Ma Y., Li X., Wang L. (2021). Dietary patterns and cardiometabolic risks in diverse less-developed ethnic minority regions: Results from the China Multi-Ethnic Cohort (CMEC) Study. Lancet Reg. Health West. Pac..

[5] Appel L.J., Moore T.J., Obarzanek E., Vollmer W.M., Svetkey L.P., Sacks F.M., Bray G.A., Vogt T.M., Cutler J.A., Windhauser M.M. (1997). A clinical trial of the effects of dietary patterns on blood pressure. DASH Collaborative Research Group. N. Engl. J. Med..

[6] Chiavaroli L., Viguiliouk E., Nishi S.K., Blanco Mejia S., Rahelić D., Kahleová H., Salas-Salvadó J., Kendall C.W., Sievenpiper J.L. (2019). DASH Dietary Pattern and Cardiometabolic Outcomes: An Umbrella Review of Systematic Reviews and Meta-Analyses. Nutrients.

[7] Talaei M., Koh W.P., Yuan J.M., van Dam R.M. (2019). DASH Dietary Pattern, Mediation by Mineral Intakes, and the Risk of Coronary Artery Disease and Stroke Mortality. J. Am. Heart Assoc..

[8] Soltani S., Arablou T., Jayedi A., Salehi-Abargouei A. (2020). Adherence to the dietary approaches to stop hypertension (DASH) diet in relation to all-cause and cause-specific mortality: A systematic review and dose-response meta-analysis of prospective cohort studies. Nutr. J..

[9] Mertens E., Markey O., Geleijnse J.M., Lovegrove J.A., Givens D.I. (2018). Adherence to a healthy diet in relation to cardiovascular incidence and risk markers: Evidence from the Caerphilly Prospective Study. Eur. J. Nutr..

[10] Feng Q., Fan S., Wu Y., Zhou D., Zhao R., Liu M., Song Y. (2018). Adherence to the dietary approaches to stop hypertension diet and risk of stroke: A meta-analysis of prospective studies. Medicine.

[11] Ao Z., Huang Z., Liu H. (2022). Spicy Food and Chili Peppers and Multiple Health Outcomes: Umbrella Review. Mol. Nutr. Food Res..

[12] Ofori-Asenso R., Mohsenpour M.A., Nouri M., Faghih S., Liew D., Mazidi M. (2021). Association of Spicy Chilli Food Consumption With Cardiovascular and All-Cause Mortality: A Meta-Analysis of Prospective Cohort Studies. Angiology.

[13] Dong X., Li Y., Yang K., Zhang L., Xue Y., Yu S., Liu X., Tu R., Qiao D., Luo Z. (2021). Associations of spicy food flavour and intake frequency with blood lipid levels and risk of abnormal serum lipid levels in Chinese rural population: A cross-sectional study. Public Health Nutr.

[14] Batiha G.E., Alqahtani A., Ojo O.A., Shaheen H.M., Wasef L., Elzeiny M., Ismail M., Shalaby M., Murata T., Zaragoza-Bastida A. (2020). Biological Properties, Bioactive Constituents, and Pharmacokinetics of Some *Capsicum* spp. and Capsaicinoids. Int. J. Mol. Sci..

[15] Olatunji T.L., Afolayan A.J. (2018). The suitability of chili pepper (*Capsicum annuum* L.) for alleviating human micronutrient dietary deficiencies: A review. Food Sci. Nutr..

[16] Amini M.R., Sheikhhossein F., Bazshahi E., Hajiaqaei M., Shafie A., Shahinfar H., Azizi N., Eghbaljoo Gharehgheshlaghi H., Naghshi S., Fathipour R.B. (2021). The effects of capsinoids and fermented red pepper paste supplementation on blood pressure: A systematic review and meta-analysis of randomized controlled trials. Clin. Nutr..

[17] Kelava L., Nemeth D., Hegyi P., Keringer P., Kovacs D.K., Balasko M., Solymar M., Pakai E., Rumbus Z., Garami A. (2022). Dietary supplementation of transient receptor potential vanilloid-1 channel agonists reduces serum total cholesterol level: A meta-analysis of controlled human trials. Crit. Rev. Food Sci. Nutr..

[18] Shi Z., Riley M., Brown A., Page A. (2018). Chilli intake is inversely associated with hypertension among adults. Clin. Nutr. ESPEN.

[19] Jang H.H., Lee J., Lee S.H., Lee Y.M. (2020). Effects of Capsicum annuum supplementation on the components of metabolic syndrome: A systematic review and meta-analysis. Sci. Rep..

[20] Sanati S., Razavi B.M., Hosseinzadeh H. (2018). A review of the effects of Capsicum annuum L. and its constituent, capsaicin, in metabolic syndrome. Iran. J. Basic Med. Sci..

[21] Yang X., Tang W., Mao D., Liu X., Qian W., Dai Y., Chen L., Ding X. (2022). Spicy food consumption is associated with abdominal obesity among Chinese Han population aged 30-79 years in the Sichuan Basin: A population-based cross-sectional study. BMC Public Health.

[22] Luo Q., Ding R., Chen L., Bu X., Xiao M., Liu X., Wu Y., Xu J., Tang W., Qiu J. (2022). The Association Between Spicy Food Intake and Risk of Hyperuricemia Among Chinese Adults. Front. Public Health.

[23] Ding X., Jiao Y., Mao D., Xu J., Tang W., Jiang B. (2021). Epidemic characteristics of stroke mortality in Chongqing. Public Health Prev. Med..

[24] Zhao X., Hong F., Yin J., Tang W., Zhang G., Liang X., Li J., Cui C., Li X., China Multi-Ethnic Cohort Collaborative Group (2021). Cohort Profile: The China Multi-Ethnic Cohort (CMEC) study. Int. J. Epidemiol..

[25] WHO (2004). ICD-10: International Statistical Classification of Diseases and Related Health Problems: Tenth Revision.

[26] Chan W.C., Millwood I.Y., Kartsonaki C., Du H., Guo Y., Chen Y., Bian Z., Walters R.G., Lv J., He P. (2021). Spicy food consumption and risk of gastrointestinal-tract cancers: Findings from the China Kadoorie Biobank. Int. J. Epidemiol..

[27] Chiu S., Bergeron N., Williams P.T., Bray G.A., Sutherland B., Krauss R.M. (2016). Comparison of the DASH (Dietary Approaches to Stop Hypertension) diet and a higher-fat DASH diet on blood pressure and lipids and lipoproteins: A randomized controlled trial1–3. Am. J. Clin. Nutr..

[28] Fung T.T., Chiuve S.E., McCullough M.L., Rexrode K.M., Logroscino G., Hu F.B. (2008). Adherence to a DASH-style diet and risk of coronary heart disease and stroke in women. Arch. Intern. Med..

[29] Ao L., Zhou J., Han M., Li H., Li Y., Pan Y., Chen J., Xie X., Jiang Y., Wei J. (2022). The joint effects of physical activity and air pollution on type 2 diabetes in older adults. BMC Geriatr..

[30] (2013). Criteria of Weight for Adults.

[31] China Hypertension Prevention Guideline Revision Committee (2019). Chinese Guidelines for the Prevention and Treatment of Hypertension (2018 Revision). Chin. J. Cardiol..

[32] Chinese Joint Committee on the Revision of Guidelines for Prevention and Treatment of Adult Dyslipidemia (2016). Guidelines for prevention and treatment of dyslipidemia in Chinese adults (2016 version). Chin. Circ. J..

[33] American Diabetes Association (2017). 2. Classification and Diagnosis of Diabetes. Diabetes Care.

[34] Andersson T., Alfredsson L., Källberg H., Zdravkovic S., Ahlbom A. (2005). Calculating measures of biological interaction. Eur. J. Epidemiol..

[35] Bonaccio M., Di Castelnuovo A., Costanzo S., Ruggiero E., De Curtis A., Persichillo M., Tabolacci C., Facchiano F., Cerletti C., Donati M.B. (2019). Chili Pepper Consumption and Mortality in Italian Adults. J. Am. Coll. Cardiol..

[36] Chopan M., Littenberg B. (2017). The Association of Hot Red Chili Pepper Consumption and Mortality: A Large Population-Based Cohort Study. PLoS ONE.

[37] Lv J., Qi L., Yu C., Yang L., Guo Y., Chen Y., Bian Z., Sun D., Du J., Ge P. (2015). Consumption of spicy foods and total and cause-specific mortality: Population based cohort study. BMJ.

[38] Ma Y., He F.J., Sun Q., Yuan C., Kieneker L.M., Curhan G.C., MacGregor G.A., Bakker S.J.L., Campbell N.R.C., Wang M. (2022). 24-Hour Urinary Sodium and Potassium Excretion and Cardiovascular Risk. N. Engl. J. Med..

[39] Govindarajan V.S., Salzer U.J. (1986). Capsicum—Production, technology, chemistry, and quality—Part II. Processed products, standards, world production and trade. Crit. Rev. Food Sci. Nutr..

[40] Rosca A.E., Iesanu M.I., Zahiu C.D.M., Voiculescu S.E., Paslaru A.C., Zagrean A.M. (2020). Capsaicin and Gut Microbiota in Health and Disease. Molecules.

[41] Wang S., Cheng L., He S., Xie D. (2019). Regional Pungency Degree in China and Its Correlation with Typical Climate Factors. J. Food Sci..

[42] Zhao Z., Li M., Li C., Wang T., Xu Y., Zhan Z., Dong W., Shen Z., Xu M., Lu J. (2020). Dietary preferences and diabetic risk in China: A large-scale nationwide Internet data-based study. J. Diabetes.

[43] Azadbakht L., Mirmiran P., Esmaillzadeh A., Azizi T., Azizi F. (2005). Beneficial effects of a Dietary Approaches to Stop Hypertension eating plan on features of the metabolic syndrome. Diabetes Care.

[44] Pérez-Cano F.J., Castell M. (2016). Flavonoids, Inflammation and Immune System. Nutrients.

[45] Reynolds A.N. (2018). Associations of fats and carbohydrates with cardiovascular disease and mortality-PURE and simple?. Lancet.

[46] Castro-Barquero S., Ruiz-León A.M., Sierra-Pérez M., Estruch R., Casas R. (2020). Dietary Strategies for Metabolic. Nutrients.

[47] Hébert J.R., Shivappa N., Wirth M.D., Hussey J.R., Hurley T.G. (2019). Perspective: The Dietary Inflammatory Index (DII)-Lessons Learned, Improvements Made, and Future Directions. Adv. Nutr..

